# Systematic study of physicochemical and electrochemical properties of carbon nanomaterials

**DOI:** 10.1039/d2ra02533g

**Published:** 2022-05-23

**Authors:** Hilal Ahmad, Rais Ahmad Khan, Bon Heun Koo, Ali Alsalme

**Affiliations:** Division of Computational Physics, Institute for Computational Science, Ton Duc Thang University Ho Chi Minh City 700000 Vietnam aalsalme@ksu.edu.sa; Faculty of Applied Sciences, Ton Duc Thang University Ho Chi Minh City 700000 Vietnam; Department of Chemistry, College of Science, King Saud University Riyadh 11451 Kingdom of Saudi Arabia; School of Materials Science and Engineering, Changwon National University Changwon 51140 Gyeongnam South Korea

## Abstract

Carbon nanomaterials exhibit exceptional properties and broad horizon applications, where graphene is one of the most popular allotropes of this family due to its astounding performance in every stratum vis-à-vis other classical materials. The large surface area of 2630 m^2^ g^−1^, high electrical conductivity, and electron mobility of non-toxic graphene nanomaterials serve as the building blocks for supercapacitor studies. In this article, comparative studies are carried out between electrochemically exfoliated graphene sheets (GSs), solvothermally synthesized graphene quantum dots (GQDs) and acid refluxed carbon nanotubes (CNTs) as an energy storage electrode nanomaterial through cyclic voltammetry (CV). The electrochemical properties of the materials are well correlated with the physicochemical characteristics obtained from Raman, Fourier-transform infrared, and absorption spectroscopy. Thin GSs (0.8–1 nm) and small size (6–10 nm) GQDs fabricated by using laboratory-grade 99% purity graphite rods resulted in promising low-cost materials at mass scale as compared to conducting CNTs. The 0D graphene quantum dots proved to be an excellent energy electrode material in an alkaline electrolyte solution compared to other carbon nanomaterials. The distinct characteristic features of GQDs, like superior electrical properties, large surface area, and abundant active sites make them an ideal candidate for utilization in supercapacitors. The GQDs exhibited an enhanced specific capacitance of 113 F g^−1^ in 6 mol L^−1^ KOH through cyclic voltammetry.

## Abstract 2

Carbon nanomaterials exhibit exceptional properties and broad horizon applications, where graphene is one of the most popular allotropes of this family due to its astounding performance in every stratum vis-à-vis other classical materials.

## Graphical abstract

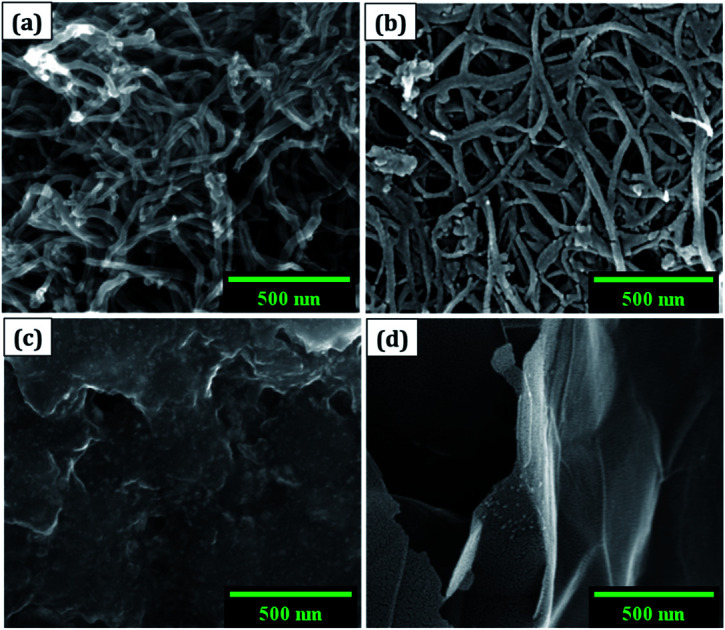

## 1. Introduction

Supercapacitors (SCs), also known as ultracapacitors, are capacitors that store electrostatic charges on the surface of electrodes and have lower energy density than batteries. The energy density stored in SCs can be improved by increasing the specific capacitance upon the application of a particular voltage.^1,2^ Further, the specific capacitance can be increased by developing a suitable nanomaterial.

Carbonous materials like carbon nanotubes (CNT), carbon aerogels, graphene nanomaterials, activated carbon, carbon nanofibers, and many more^1–3^ are prime electrode materials for electric double-layer capacitors (EDLCs). The working principle of EDLCs involves non-faradaic reactions occurring on the electric double layer.^4,5^ The enhanced properties like large surface area, high conductivity, and cost-effectiveness are attributed to their vast use in the field of energy storage devices.^6^ The drawback associated with most carbonaceous nanomaterials is their hydrophobic nature which results in the agglomeration and non-uniform dispersion in any solvent.^7^ This leads to poor supercapacitance performance and causes hindrance to many other promising applications.^8^ Besides EDLCs, another branch of supercapacitors are the pseudocapacitors that operate on the faradaic reactions arising at the electrode material surface like metal oxides, conducting polymers, and their composites with carbon nanomaterials.^9^

Multi-walled carbon nanotubes (MWCNTs) and single-walled carbon nanotubes (SWCNTs) are two types of carbon nanotubes (SWCNTs). MWCNTs are built up from a series of coaxial cylinders, each of which is made up of a single graphene sheet and surrounds a hollow cone. MWCNTs have an outside diameter ranging from 2–100 nm, an interior diameter ranging from 1–3 nm, and a length ranging from one to several micrometers.^1,2^ The patterns of graphite layers in MWCNTs may be divided into two categories: one has a parchment-like structure with a graphene sheet coiled up around it, and the other is known as the Russian doll model, which has a layer of graphene sheet placed within a concentric structure. SWCNTs are made up of a single cylindrical carbon layer with a diameter ranging from 0.4 to 2 nanometers, depending on the temperature at which they were made. It was discovered that the diameter of CNTs increases as the temperature rises and SWCNTs can be arranged in an armed chair, zigzag, chiral, or helical patterns.^1,2^

Graphene is one the most important member of the carbon family due to its enhanced performance in every field.^10^ Many efforts had been made in the past to produce hydrophilic graphene sheets and quantum dots (QDs); like nitric and sulfuric acid refluxing treatment to boost capacitance and other properties.^11^ In our recent publication, we have synthesized low-cost, solution-processable, and high-quality graphene sheets (GSs) and graphene quantum dots (GQDs) using laboratory-grade graphite electrodes.^8^ GQDs are graphene sheets with quantum-scale dimensions, *i.e.*, 0D nanomaterials, with substantially larger specific surface areas and more accessible edges than graphene sheets.^7^ In this report, comparative energy storage properties of MWCNT, SWCNT, graphene, and its quantum dots are studied through electrochemical cyclic voltammetry (CV) measurements. Although the energy storage properties of carbon nanomaterials in the individual and composite forms are explored extensively in the past, systematic comparison drawn amongst them is scarcely reported in the literature. In addition, electrochemical features are correlated with the morphological patterns, solubility, structural defects, and oxygen functionalities of carbon materials. There are many reports available implicating graphene nanomaterials to be better energy electrode materials than CNTs, but relative comparative study with in-depth reasoning is rather scarce in the literature.

## 2. Experimental

### 2.1. Reagents and solutions

All chemicals used were of analytical reagent grade and double distilled (DI) water was used to make the solutions. Platinum (Pt) mesh, reference electrode Ag/AgCl [3 mol L^−1^ KCl] and polyvinylidene fluoride (PVDF) from Sigma-Aldrich; laboratory-grade graphite rod [99.9% pure] from local vendor; 0.22 μm membrane filter and dialysis membrane-50 (LA387-10MT) from Whatman and Himedia; *N*-methyl-2-pyrrolidone (NMP), nitric acid (HNO_3_), dimethylformamide (DMF) and ethylenediamine (en) from Thermo Fisher Scientific; potassium hydroxide pellets (KOH), sodium chloride (NaCl) salt and *ortho*-phosphoric acid (H_3_PO_4_) were procured from Merck, India. Single-walled carbon nanotubes (SWCNTs) (99% pure, P2 variety) from Carbon Solutions Inc., and multi-walled carbon nanotubes (MWCNTs) from Chengdu Organic Chemicals Co., China were purchased.

### 2.2. Fabrication details

The purchased SWCNTs and MWCNTs powder were refluxed in 5 mol L^−1^ HNO_3_ for 5 h at 100 °C to acid functionalize and remove unwanted metal impurities.^12^ Further, washing was performed through ultrasonication and centrifugation with DI water and ethanol to remove acid and bring the pH neutral. Many methods are reported in the past to obtain high-quality GQDs, showing enhanced properties than carbon-quantum dots.^13–16^ In addition, graphene sheets and quantum dots were prepared according to the details published in our earlier report.^8^ The protocol followed for energy storage measurements is also discussed below. Briefly:

(a) Graphene sheets (GSs): graphite rod (anode) was exfoliated in an electrolytic cell with Pt mesh as a cathode in 0.5 mol L^−1^ NaCl electrolyte at room temperature (RT) under constant DC potential ranging from 2–10 V using Solartron 1280C. The obtained product solution was rinsed completely to obtain neutral pH, sonicated for 2 h to get homogenous graphene suspension, and filtered through a 0.22 μm filter membrane for thin GSs.

(b) Purified graphene sheets (PGS): the above derived GSs (50 mg) were refluxed in 5 mol L^−1^ HNO_3_ acid at 100 °C for 24 h under continuous stirring to purify and reduce the lateral flake size. The suspension was washed thoroughly to remove the acid and bring the pH neutral.

(c) Graphene quantum dots (GQDs): the acquired diminished size PGS was suspended in a mixture of 5 mol L^−1^ H_3_PO_4_ (20 mL, protic acid) and 30 mL ethylenediamine. The suspension was probe sonicated for 2 h and transferred to a 100 mL Teflon lined stainless steel autoclave, heated at 200 °C for 8 h. The resultant consisted of black sediment carrying larger graphene particles with brown supernatant as GQDs. The larger graphene nanoparticles were separated by vacuum filtration with a 0.22 μm filter membrane and dialysing the collected filtrate over DI water in a dialysis bag for 24 h to obtain GQDs.

(d) Cyclic voltammetry (CV) measurements: the energy-related electrochemical studies were carried out using the three-electrode system with MWCNT, SWCNT, GQDs, and PGS as working electrodes (WE), platinum wire as a counter electrode (CE), and Ag/AgCl as reference electrode (RE), respectively using Solartron 1280C. Further, WEs were fabricated by dissolving 90 wt% (1 g) active material in a mixture of 10 wt% (0.1 g) PVDF (adhesive) and 100 μL NMP (dispersion medium) creating a free-standing film (1 × 1 cm^2^, Fig. 3f) using a doctor's blade, with an oven baking at 60 °C for 24 h. All the CV experiments were carried out in an alkaline aqueous solution of 6 mol L^−1^ KOH at room temperature (RT).

## 3. Results and discussions

The properties of the synthesized graphene nanomaterials are already discussed in length in our previous publication.^8^ In order to carry out systematic equivalence, the structural properties are measured again for comparative analysis of different carbon nanostructures, as discussed below.

### 3.1. Physicochemical characterization

**Fig. 1:**
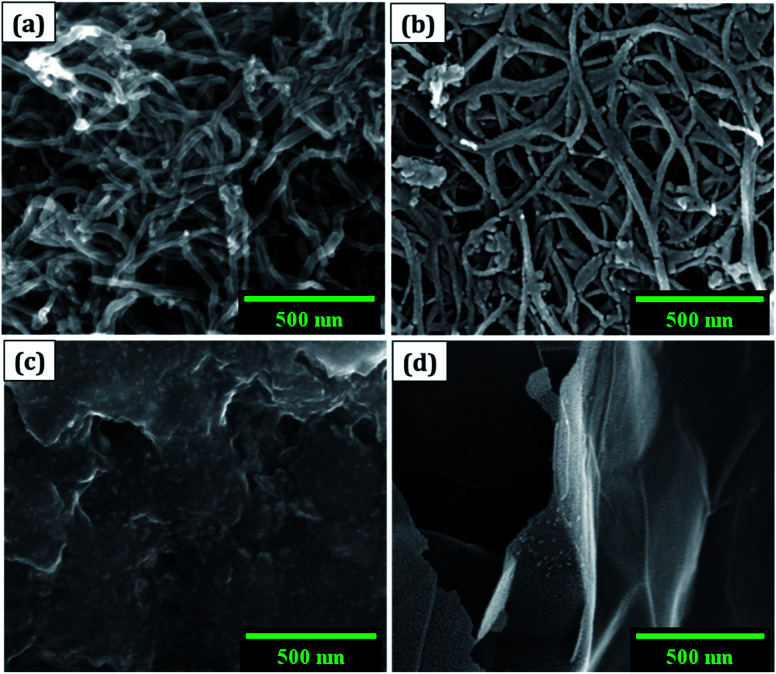
FESEM images of (a) MWCNT, (b) SWCNT, (c) GQDs, and (d) PGS.

**Fig. 2:**
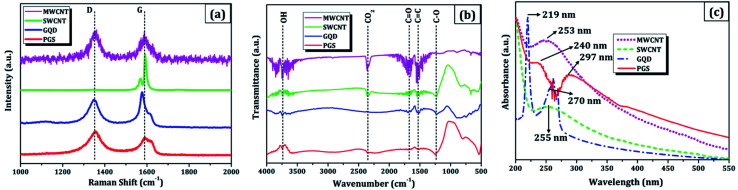
Combined physicochemical properties of carbon nanomaterials: (a) Raman spectra, (b) FTIR plot, and (c) UV-vis spectrograph.

(a) The morphology was observed using a field emission scanning electron microscope (FESEM: Nova NanoSEM 450, FEI). The cluster of SWCNT and MWCNT possess diameters of 2–5 nm and 2–80 nm with lengths in micrometres, respectively as illustrated in Fig. 1a and b. The size of the electrochemically exfoliated graphene sheets in NaCl electrolyte was reduced to ∼1 μm by refluxing in nitric acid yielding silk and a smooth texture PGS (Fig. 1d). The size of the sheets was further diminished to 6–10 nm by the formation of GQDs through the solvothermal method as shown in Fig. 1c, where GQDs are decorated on the graphene sheets. The FESEM micrograph of GQDs indicated below is before dialysis separation procedure from the sheets.

(b) A micro-Raman spectrometer (Lab RAM HR 800, Horiba) was used to produce a Raman spectrum through an Olympus 100× objective lens with a spot size of 1.19 μm, an excitation wavelength of 514 nm, and a 4 mW optical power of an argon-ion laser. The D band (∼1353 cm^−1^) is related to disordered structural defects (*e.g.*, amorphous carbon or edges that potentially break the symmetry and selection rule),^17–19^ while the G band (∼1584 cm^−1^) is connected with the first-order scattering of the E_2g_ mode found for sp^2^ carbon domains. *I*_D_/*I*_G_ (sp^3^/sp^2^) is the integrated area ratio, a small value of which signifies low defects and high crystallinity in the carbonaceous sample. The defect density was reduced in GQDs (*I*_D_/*I*_G_ ∼ 0.78) obtained after the high temperature and pressure treatment on PGS (*I*_D_/*I*_G_ ∼ 1.32) as shown in Fig. 2a. The defect density is low in MWCNTs with *I*_D_/*I*_G_ ∼ 1 and ∼zero defects in SWCNTs due to fewer oxygen functional groups attached during chemical treatment. SWCNTs, GQDs, and PGS all have tangential (G-band) multi-feature at higher frequencies, but in the case of current MWCNTs, it indicates that the innermost nanotube diameter is less than 2 nm.^1,2^

**Fig. 3:**
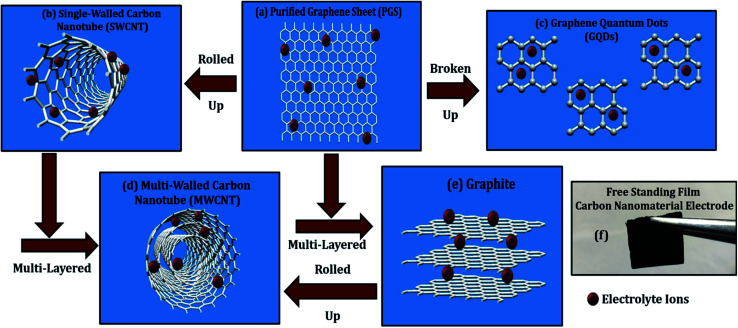
Comparison of discussed carbon nanomaterials in this article as electrode material for supercapacitors: (a) graphene (PGS), (b) SWCNTs, (c) GQDs, (d) MWCNTs, (e) graphite, and (f) camera image of the energy electrode free-standing film.

(c) Fourier-transform infrared spectroscopy (FTIR, Vertex70 V, Bruker Optik) was used to investigate the functionalized groups attached to the carbon nanomaterial pellets. The FTIR spectra (Fig. 2b) of MWCNT, SWCNT, GQDs, and PGS consist of different oxygen functionalities such as O–H stretching (hydroxyl group: 3300–3800 cm^−1^), C=O (carboxyl group: 1670–1710 cm^−1^), C–O–C stretching (epoxy group: 1080–1255 cm^−1^), C=C (sp^2^ hybridised: 1500–1600 cm^−1^), CO_2_ (2300–2400 cm^−1^) and these make them soluble in solvents. The presence of an intense peak of CO_2_ group can also be ascribed to the sample contamination. The ionisation of abundant oxygen groups usually results in GQDs aqueous dispersion being quite stable.^20^ The graphene sheets (PGS) possess an increased number of oxygen functional groups connected to them after reflux treatment vis-à-vis source graphene sheets (GSs). The dispersion of virgin SWCNTs and MWCNTs in water, on the other hand, is unstable and tends to cluster, therefore the CNTs were acid refluxed prior to the use in an application.^1,2^

(d) Absorption spectra were obtained using a UV-visible spectrophotometer (UV-vis, Cary 100, Agilent) with a path length of 1 cm and a scan rate of 120 nm min^−1^. In all cases, the concentration of nanomaterial dispersed was 0.5 mg mL^−1^ in DMF solvent through bath sonication for 0.5 h. The enhanced solubility in water and other solvents can be attributed to oxygen-containing functionalities (hydroxyl, carbonyl, carboxyl) decorated on the carbon materials as studied in FTIR analysis.^21,22^ The degree of residual conjugation (π–π* transition) is determined by the peak around 227–231 nm range and n–π* transition of carbonyl groups is responsible for the shoulder at about 300 nm. Both the absorption peaks are observed in graphene nanomaterials (PGS and GQDs) and in the case of CNTs, the structural peaks can be seen around 250 nm (Fig. 2c). These oxygen-containing functional groups have the potential to benefit a variety of applications, primarily because they may be utilised to add multifunctionalities, which are responsible for carbon nanomaterials' solubility in water and other solvents.^23^

### 3.2. Electrochemical characterization

Due to its enormous surface area and inexpensive cost, activated carbon is the most often utilised electrode material for supercapacitors. Low electrical conductivity and fewer electrochemical active sites, however, restrict its use in supercapacitors with high power density.^24^ Only the outermost section of SWCNTs (Fig. 1b) can operate for ion absorption, while the interior carbon atoms are entirely squandered. MWCNTs are more prone to stack in bundles (Fig. 1d), resulting in reduced specific capacitance in CNT-based supercapacitors. Graphene has attracted a lot of attention as an energy electrode material for lithium-ion batteries and supercapacitor with Stoller *et al.*^25^ reporting capacitances of 135 F g^−1^. Capacitances of 197.2 F g^−1^ were recorded in graphene oxide (GO)^26,27^ and metal oxide composites such ZnO^28^ and MnO_2_.^29^ Graphene nanosheets (Fig. 1a) are likely to clump together during the drying process to create graphite due to van der Waals interactions (Fig. 1e). Electrolyte ions would have a hard time getting into the ultrasmall pores, especially bigger ions like an organic electrolyte or at a rapid charging rate.^30,31^ Furthermore, the manufacturing of binder-free supercapacitor electrodes, such as graphene paper electrodes, is a typical technique for reducing the contact resistance between graphene nanosheets and the current collector for supercapacitors with high power characteristics.^30^ However, due to the densely packed structure of graphene paper, the barrier between graphene and the electrolyte lowers the conductivity of graphene materials, and the problem continues.^32,33^ In aqueous NaCl electrolyte, rGO sheets had a specific capacitance of 75.29 F g^−1^.^34^ In addition, in a 6 mol L^−1^ KOH electrolyte, carbon quantum dots (CQDs) assembled to a multilayer carbon demonstrated volumetric capacitance of 157.4 F cm^−3^.^35^ Breaking the nanosheets into graphene quantum dots (Fig. 1c) to boost the specific capacitance is another way to address the above-mentioned difficulties. In addition, alkaline aqueous solution KOH is chosen as an electrolyte majorly due to its high ionic conductivity, high mobility of OH^−^ ions in water solutions, and easy penetration into the electrode surface because of its small size.^8^ Further, a high concentration of 6 mol L^−1^ is used for increasing the number of ions and ionic conductivity of the electrolyte. As a result, such solutions are as conducting as strong acidic electrolytes, therefore serving as another solution to enhance capacitance.^36,37^

**Fig. 4:**
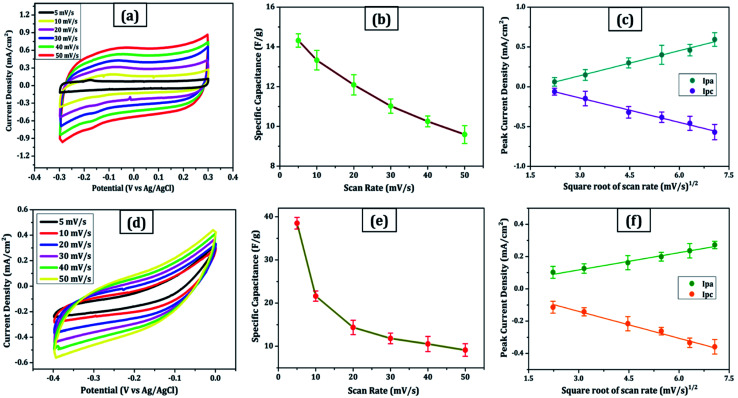
MWCNT: (a) cyclic voltammetry scans were recorded at 5–50 mV s^−1^ in 6 mol L^−1^ KOH, (b) specific capacitance *versus* scan rate plot, (c) peak current as a function of the square root of scan rate. (d–f) Similar plots in the same sequence for SWCNT.

**Fig. 5:**
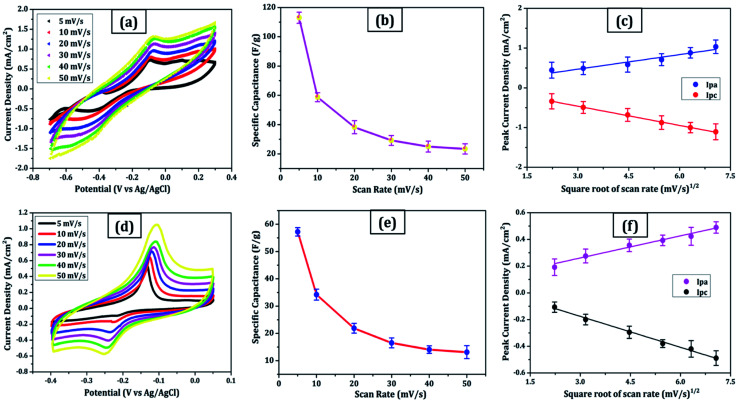
GQDs: (a) cyclic voltammetry scans were recorded at 5–50 mV s^−1^ in 6 mol L^−1^ KOH, (b) specific capacitance *versus* scan rate plot, (c) peak current as a function of the square root of scan rate. (d–f) Similar plots in the same sequence for PGS.

For comparison, CVs were collected for all the carbon electrodes under identical experimental circumstances as mentioned in Section 2.2(d). These researches assisted in the knowledge of the link between the structural and electrochemical characteristics of carbon-based electrodes, pointing to the possible use of carbon nanomaterial films in biosensing, energy conversion, biomedical, and other electronic systems.^38^ In the edge and basal planes, which are critical to the behaviour of graphitic electrodes, chemical and electrochemical reactivities differ dramatically. Furthermore, edge plane-like flaws at their basal planes are called defect sites, which might influence the electron structure and hence the density of electronic states (DOS). CV experiments were performed within a potential window of −0.3 to +0.3 V, 0 to −0.4 V, −0.7 to + 0.3 V, and −0.4 to +0.05 V *vs.* Ag/AgCl for MWCNT, SWCNT, GQDs, and PGS, respectively (Fig. 4(a and d) and 5(a and d)).

The rectangular shaped voltammogram with large current separation between the forward and reverse scans, symmetric in both anodic and cathodic directions indicates electrochemical double layer capacitive behaviour^1,2^ as observed in all carbon nanomaterials. EDLCs have some problems including unsatisfactory low specific energy density and poor rate performance due to their limited accessible storage sites. Although a pseudocapacitive contribution is also present, represented by faradaic redox signals where current changes upon voltage scan.^39,40^ The capacitive nature remains preserved even at higher scan rates with a great current density as observed in Fig. 4(a and d) and 5(a and d), indicating good electrochemical activity arising out of the higher electrochemical active surface area and high-power density values for carbon materials employed as electrode material. The high solubility of the treated carbon nanomaterials, as observed from the UV-visible spectra (Fig. 2c) marks the fabrication of properly scattered electrodes. Capacitance increases with decreasing scan rate as shown in Fig. 4(b and e) and 5(b and e). The CV suggested no peak formation in pure KOH electrolyte dictating a pure electric-double layer capacitive behaviour in the case of CNTs (MWCNTs and SWCNTs) and pseudocapacitive nature in GQDs. The abundance of oxygen functional groups on the graphene sheets (PGS) can be reduced and regenerated electrochemically which provides it with an inherent redox activity. The specific capacitance (*C*_s_ in F g^−1^) was determined using the equation below:^41^

1 *C*_s_ = *Q*/*m*Δ*V*,

where *Q* is the average charge during a complete redox reaction, *m* is the mass of active material and Δ*V* is the potential window. The ratio of the cathodic peak current (*I*_pc_) to the anodic peak current (*I*_ac_) is ∼1, indicative of comparatively swift, electrochemically quasi-reversible electron transfer kinetics. Anodic current increases directly with increasing square root of the scan rate,^41^ as shown in Fig. 4(c and f) and 5(c and f).

The relationship between the scan rate and peak current was obtained through linear fitting analysis in the form of a regression equation drawn below:

(a) Fig. 4c:

2 *I*_pa_ (mA) = 0.10*x* + 0.175 (*R*^2^ = 0.996)

3 *I*_pc_ (mA) = −0.10*x* + 0.165 (*R*^2^ = 0.991)

(b) Fig. 4f:

4 *I*_pa_ (mA) = 0.035*x* + 0.014 (*R*^2^ = 0.983)

5 *I*_pc_ (mA) = −0.056*x* + 0.027 (*R*^2^ = 0.945)

(c) Fig. 5c:

6 *I*_pa_ (mA) = 0.12*x* + 0.116 (*R*^2^ = 0.969)

7 *I*_pc_ (mA) = −0.16*x* + 0.019 (*R*^2^ = 0.998)

(d) Fig. 5f:

8 *I*_pa_ (mA) = 0.056*x* + 0.093 (*R*^2^ = 0.987)

9 *I*_pc_ (mA) = −0.077*x* + 0.055 (*R*^2^ = 0.993)

These results are in line with the literature's prediction of 14 to 80 F g^−1^ for SWCNTs and MWCNTs,^1,2^ which is lower than graphene despite the former's higher conductivity; due to random orientation, and bundling of the former. The CNTs electrode showed no peaks, as one would anticipate from a carbon material that exhibits the double layer capacitor behaviour. Graphene sheets are extremely electroactive and have rapid electron transfer kinetics due to their unique structural and electrical characteristics.^5^ The unusual electrical structure of PGS is responsible for the quick heterogeneous electron kinetics between the electrode and the solution. At normal temperature, the graphene sheet behaves as a semimetal or a zero-bandgap semiconductor with extraordinarily high electron mobility, which might improve electron transport when its plane is directly in touch with the electrolyte.^27^ In contrast, monolayer GQDs showed high surface area, uniform size distribution, and great porosity resulting in high specific capacitance vis-à-vis graphene sheets and CNTs.

The electrochemical exfoliation for forming GSs at a relatively high voltage of 10 V has effectively inspired the deoxygenation of functional groups along with GSs.^8^ In sample PGS, more oxygen functionalities are decorated as compared to source GS due to acid reflux therapy. Further, the synthesis of GQDs in the oxidative acid hydrothermal atmosphere resulted in a combination of monolayer and few-layer GQDs. Deoxygenation and reduction arise due to the pressure induced during the hydrothermal heating process and oxygen functional groups are introduced on the edges because of the presence of protic acid H_3_PO_4_ (aqueous) as solvent. The stated observations are confirmed with FTIR analysis described in Section 3.1(c). The electron transmission process on graphitic materials is greatly accelerated by the presence of edge-plane imperfections.^7^ The material with the high D/G ratio obtained from Raman spectra (Fig. 2a) offers the fastest electron transfer, as observed in graphene quantum dots having the greatest capacity for storing charge; making them ideal capacitors. C/O atomic% ratio and the number of layers are derived from the Energy Dispersive X-ray (EDX, QUANTAX X 129 eV, Bruker) and Raman spectra, respectively for each carbon nanomaterial as mentioned in Table 1. The EDX spectra revealed no other elements other than carbon and oxygen, signifying the purity of carbon nanomaterials.^8^

**Table 1:** Comparative list describing the number of layers, C/O atomic% ratio, and corresponding specific capacitance of each carbon nanomaterial

S. no.	Carbon nanostructure	No. of layers	C/O atomic%	Specific capacitance (F g^−1^)
1	Multi-walled carbon nanotubes (MWCNT)	Multi-layers	19	14
2	Single-walled carbon nanotubes (SWCNT)	Monolayer	24	38
3	Graphene quantum dots (GQD)	Monolayer and few layers	2.30	113
4	Purified graphene sheets (PGS)	Few layers	4.02	57

## 4. Conclusion

The high structural purity and enhanced solubility revealed excellent specific capacitance of GQDs vis-à-vis PGS, MWCNT, and SWCNT. This implies that the high defect density, large surface area, and high porosity of graphene quantum dots result in superior specific capacitance, thereby proving to be a better energy electrode nanomaterial candidate. GQDs showed higher current density and capacitance vis-à-vis other carbon materials in the solutions indicating promising materials for supercapacitors and lithium-ion batteries. Such energy storage materials are proving to be a boon for electronic device technology. In addition, this paper demonstrated intra-comparison between the carbon nanomaterials rather than differentiating it from the data reported in other articles.

## Conflicts of interest

There are no conflicts to declare.

## Supplementary Material

## References

[cit1] Aboutalebi S. H. (2011). *et al.*, Comparison of GO, GO/MWCNTs composite and MWCNTs as potential electrode materials for supercapacitors. Energy Environ. Sci..

[cit2] Azam M. A., Fujiwara A., Shimoda T. (2013). Significant capacitance performance of vertically aligned single-walled carbon nanotube supercapacitor by varying potassium hydroxide concentration. Int. J. Electrochem. Sci..

[cit3] Tang L. (2009). *et al.*, Preparation, structure, and electrochemical properties of reduced graphene sheet films. Adv. Funct. Mater..

[cit4] Krishnamoorthy K. (2014). *et al.*, Plasma assisted synthesis of graphene nanosheets and their supercapacitor applications. Sci. Adv. Mater..

[cit5] Brownson D. A. C. (2014). *et al.*, Electrochemical properties of CVD grown pristine graphene: monolayer-vs. quasi-graphene. Nanoscale.

[cit6] Fan Z. (2012). *et al.*, Easy synthesis of porous graphene nanosheets and their use in supercapacitors. Carbon.

[cit7] Zhang S. (2018). *et al.*, High-performance supercapacitor of graphene quantum dots with uniform sizes. ACS Appl. Mater. Interfaces.

[cit8] Ahmad H. (2021). *et al.*, Ultra-thin graphene oxide membrane deposited on highly porous anodizedaluminum oxide surface for heavy metal ions preconcentration. J. Hazard. Mater..

[cit9] Chidembo A. T. (2010). *et al.*, Nickel (II) tetra-aminophthalocyanine modified MWCNTs as potential nanocomposite materials for the development of supercapacitors. Energy Environ. Sci..

[cit10] Le Fevre L. W. (2019). *et al.*, Systematic comparison of graphene materials for supercapacitor electrodes. ChemistryOpen.

[cit11] Muthurasu A., Dhandapani P., Ganesh V. (2016). Facile and simultaneous synthesis of graphene quantum dots and reduced graphene oxide for bio-imaging and supercapacitor applications. New J. Chem..

[cit12] Canobre S. C. (2009). *et al.*, Synthesis and characterization of hybrid composites based on carbon nanotubes. Electrochim. Acta.

[cit13] Hou Y. (2015). *et al.*, One-pot electrochemical synthesis of functionalized fluorescent carbon dots and their selective sensing for mercury ion. Anal. Chim. Acta.

[cit14] Kandasamy G. (2019). Recent advancements in doped/co-doped carbon quantum dots for multi-potential applications. C.

[cit15] Devi N. R., Kumar T. H. V., Sundramoorthy A. K. (2018). Electrochemically exfoliated carbon quantum dots modified electrodes for detection of dopamine neurotransmitter. J. Electrochem. Soc..

[cit16] Devi S. (2018). *et al.*, Ethylenediamine mediated luminescence enhancement of pollutant derivatized carbon quantum dots for intracellular trinitrotoluene detection: soot to shine. RSC Adv..

[cit17] Wu J.-B. (2018). *et al.*, Raman spectroscopy of graphene-based materials and its applications in related devices. Chem. Soc. Rev..

[cit18] Cheng H. (2012). *et al.*, Graphene-quantum-dot assembled nanotubes: a new platform for efficient Raman enhancement. ACS Nano.

[cit19] Nguyen H. Y. (2019). *et al.*, Microwave-assisted synthesis of graphene quantum dots and nitrogen-doped graphene quantum dots: Raman characterization and their optical properties. Adv. Nat. Sci.: Nanosci. Nanotechnol..

[cit20] Zhu S. (2012). *et al.*, Graphene quantum dots with controllable surface oxidation, tunable fluorescence and up-conversion emission. RSC Adv..

[cit21] Qian Z. (2013). *et al.*, Surface functionalization of graphene quantum dots with small organic molecules from photoluminescence modulation to bioimaging applications: an experimental and theoretical investigation. RSC Adv..

[cit22] Yan X. (2010). *et al.*, Large, solution-processable graphene quantum dots as light absorbers for photovoltaics. Nano Lett..

[cit23] Zhu Y. (2010). *et al.*, Graphene and graphene oxide: synthesis, properties, and applications. Adv. Mater..

[cit24] Cheng Q. (2011). *et al.*, Graphene and carbon nanotube composite electrodes for supercapacitors with ultra-high energy density. Phys. Chem. Chem. Phys..

[cit25] Stoller M. D. (2008). *et al.*, Graphene-based ultracapacitors. Nano Lett..

[cit26] Ahmad H. (2019). *et al.*, Separation and preconcentration of Pb(ii) and Cd(ii) from aqueous samples using hyperbranched polyethyleneimine-functionalized graphene oxideimmobilized polystyrene spherical adsorbents. Microchem. J..

[cit27] Ambrosi A., Martin P. (2016). Electrochemically exfoliated graphene and graphene oxide for energy storage and electrochemistry applications. Chem.–Eur. J..

[cit28] Zhang Y. (2009). *et al.*, Capacitive behavior of graphene–ZnO composite film for supercapacitors. J. Electroanal. Chem..

[cit29] Khomenko V., Raymundo-Pinero E., Béguin F. (2006). Optimisation of an asymmetric manganese oxide/activated carbon capacitor working at 2 V in aqueous medium. J. Power Sources.

[cit30] Parvez K. (2014). *et al.*, Exfoliation of graphite into graphene in aqueous solutions of inorganic salts. J. Am. Chem. Soc..

[cit31] Zhao C., Zheng W., Wang X., Zhang H., Cui X., Wang H. (2013). Ultrahigh capacitive performance from both Co(OH)_2_/graphene electrode and K_3_Fe(CN)_6_ electrolyte. Sci. Rep..

[cit32] Ahmad H. (2021). *et al.*, Graphene oxide lamellar membrane with enlarged inter-layer spacing for fast preconcentration and determination of trace metal ions. RSC Adv..

[cit33] Kim K. S. (2009). *et al.*, Large-scale pattern growth of graphene films for stretchable transparent electrodes. Nature.

[cit34] da Silva F. D. (2020). *et al.*, In situ electrochemical exfoliation of embedded graphite to superficial graphene sheets for electroanalytical purposes. Electrochim. Acta.

[cit35] Chen G. (2016). *et al.*, Assembling carbon quantum dots to a layered carbon for high-density supercapacitor electrodes. Sci. Rep..

[cit36] Imran H., Manikandan P. N., Dharuman V. (2015). Facile and green synthesis of graphene oxide by electrical exfoliation of pencil graphite and gold nanoparticle for non-enzymatic simultaneous sensing of ascorbic acid, dopamine and uric acid. RSC Adv..

[cit37] Tripathi P. (2015). *et al.*, High yield synthesis of electrolyte heating assisted electrochemically exfoliated graphene for electromagnetic interference shielding applications. RSC Adv..

[cit38] Hamdy E. (2020). *et al.*, Enhancement of Molten Nitrate Thermal Properties by Reduced Graphene Oxide and Graphene Quantum Dots. ACS Omega.

[cit39] Huang B. (2018). *et al.*, Facile synthesis of an all-in-one graphene nanosheets@ nickel electrode for high-power performance supercapacitor application. RSC Adv..

[cit40] Dreyer D. R. (2010). *et al.*, From Conception to Realization: An Historial Account of Graphene and Some Perspectives for Its Future. Angew. Chem., Int. Ed..

[cit41] Khattak A.M. (2016). A redox-active 2D covalent organic framework with pyridine moieties capable of faradaic energy storage. J. Mater. Chem. A.

